# The Current Role of Physiotherapy in Systemic Light-Chain (AL) Amyloidosis and Multiple Myeloma

**DOI:** 10.3390/life16061018

**Published:** 2026-06-17

**Authors:** Ana Ríos-Sánchez, María Angustias Riazzo-Benítez, Rafael Ríos-Tamayo

**Affiliations:** 1 Department of Physiotherapy, INBO Clinic, 18006 Granada, Spain; ana.rios.sanchez.93@gmail.com; 2 Department of Physiotherapy, Albolote Health Center, 18220 Granada, Spain; mariariazzo@gmail.com; 3 Department of Hematology, University Hospital Virgen de las Nieves, 18014 Granada, Spain; 4 Instituto de Investigación Biosanitaria ibs. GRANADA, 18012 Granada, Spain; 5 CIBER Epidemiology and Public Health, 28029 Madrid, Spain

**Keywords:** physiotherapy, systemic light-chain (AL) amyloidosis, multiple myeloma, supportive therapy, multidisciplinary clinic, efficiency, outcome, survival, health-related quality of life, referral center

## Abstract

Physiotherapy is an evidence-based healthcare occupation aiming to collaborate in the diagnosis, prevention and treatment of a myriad of diseases and clinical scenarios throughout all stages of human life. Its development has been accelerated over the last two decades. The scope of physiotherapy is continuously evolvig. However, the accumulated evidence in the context of rare diseases is scarce. Remarkably, the opportunity for improvement and potential benefit for complex diseases with low prevalence is also very high, both as an isolated approach or within multidisciplinary specialized units. Systemic light-chain (AL) amyloidosis is a rare, chronic, complex, heterogeneous, incurable, and challenging disease, which may involve different organs and systems, including the heart, kidney, liver, peripheral nerves, lung, muscle, skin, and others. Heart is the most frequently involved organ leading to failure and arrhythmias. Peripheral neuropathy is a relatively frequent symptom. Renal, respiratory, and hepatic failure may also occur. The aim of this narrative review is summarizing, updating, and critically underlining potential new avenues of development on the role of physiotherapy in systemic light-chain (AL) amyloidosis, compared with its application in multiple myeloma, a closely related but not so rare entity.

## 1. Introduction

According to the World Confederation for Physical Therapy, physiotherapy (PT) is defined as “a health profession focused on human function and movement, aiming to maximize physical potential, health-related quality of life (HRQoL), and movement within promotion, prevention, treatment, and rehabilitation, using physical approaches like exercise, manual therapy, and education to restore well-being across diverse conditions” [1]. The scope and practice of PT are changing over time and depend on the specific healthcare background, socioeconomic characteristics, and other variables of the country and health system in which it is practiced [2,3,4,5,6,7].

In summary, PT is a patient-focused and community-centered healthcare discipline that should be integrated in all levels of the healthcare system (primary care, hospital, intensive care, …) [8,9], contextualized in a socio-health environment [10], and led by evidence-based and, in recent years, artificial intelligence (AI)-driven research [11,12,13]. As with other healthcare professions, physiotherapists (PTs) have become increasingly specialized in certain areas [14].

Systemic light-chain (AL) amyloidosis is a rare entity, with crude incidence and prevalence rates of 10–15 and 40–60 cases per million population, respectively, standardized rates being somewhat lower [15]. It is classified under the category of “plasma cell neoplasms (PCN) and other diseases with paraproteins” in the fifth edition of the World Health Organization classification of lymphoid tumors [16]. The heart is the most frequently involved organ, followed by kidney, liver, nervous system, gastrointestinal tract, lungs, muscle, skin, and others. The cardiac involvement (CA) in AL amyloidosis (AL-CA) occurs in approximately 80% of patients and is the key prognostic factor in terms of overall survival (OS) and early mortality [17].

Multiple myeloma (MM) is the second most common hematological neoplasm and it is considered the prototype of PCN. Age-standardized incidence rate is higher than 5/100,000 inhabitants/year in most European countries and prevalence is dramatically rising due to increasingly prolonged OS, with crude prevalence rates generally higher than 40/100,000 inhabitants/year [18,19,20]. Therefore, the incidence of newly diagnosed (ND) MM (NDMM) is approximately five times greater than that of ND AL (NDAL) cases. On the other hand, one out of five NDAL also fulfills the current diagnostic criteria of MM. Patients with concomitant AL and MM (AL/MM) have a poor outcome. The pattern of organ involvement in MM is different from that in AL patients, with bone being the key involved organ, besides kidney. Pain and anemia symptoms are common. The risk of pathological fractures as well as infection is increased [21].

Frailty is commonly defined as a dynamic, multidimensional, potentially reversible, age-related syndrome, characterized by low physiological reserve and reduced resistance to stressors, leading to increased vulnerability to adverse outcomes [22,23,24]. Frailty can be easily assessed in both settings using a simple score based on three variables: age, Eastern Cooperative Oncology Group (ECOG) performance status (PS) (<2 vs. ≥2) and N-terminal fragment of the pro-brain natriuretic peptide (NT-proBNP) (<8500 vs. ≥8500 ng/L) [25].

Despite a lack of standardization, comorbidity is crucial in the prognosis assessment of cancer patients in general, and in patients with AL and MM in particular [25,26].

Both AL and MM are chronic, incurable, unpreventable, complex, and heterogeneous diseases. They are slightly more frequent in men, and median age at diagnosis is 60–70 years in most studies. Both diseases are characterized by a bone marrow infiltration of clonal plasma cells, but bone damage is typical of MM (and AL/MM), whereas it is exceptional in AL. Both entities show in most cases a monoclonal protein (M protein) in serum and/or urine. Anti-clonal therapy [27], including high doses of melphalan followed by autologous stem cell transplant (ASCT) in transplant-eligible (TE) patients, is the basis of the treatment. A key difference is the pattern of organ involvement, with the heart crucial in AL whereas in MM bone disease (pathological fractures, paraskeletal involvement) has a major role, besides the presence of extramedullary disease.

Exercise has been extensively recommended for cancer in different ways and contexts with variable evidence and results [28], but barriers to the appropriate integration of qualified exercise professionals (QEPs) have been identified [29]. Exercise remains the most frequently reported PT-related intervention in cancer. A tailored exercise program should be considered crucial in the management of every cancer patient, and a specific exercise prescription should be recommended for a specific entity, considering the available evidence [30,31].

AL and MM are two separate but linked PCNs that should be ideally managed in the setting of a specialized multidisciplinary unit including several medical specialties, nursing, psychologists, pharmacists, social workers, and other healthcare professionals such as PTs. The aim of this study is to critically analyze the current evidence on the role of PT in both clinical scenarios, trying to draw a landscape in which the potential benefits of applying an optimal and personalized PT approach could be highlighted, and hopefully help to bridge the gap between reality and excellence.

## 2. Materials and Methods

A systematic English PubMed search was performed until March 2026, including the following terms: “systemic light-chain (AL) amyloidosis, primary amyloidosis, cardiac amyloidosis, multiple myeloma, plasma cell neoplasm, physiotherapy, physical therapy, treatment, and therapy”. This search was complemented with other searches including specific issues of interest such as exercise, physical activity, nutrition, diet, fatigue, heart failure, autologous stem cell transplant, renal failure, neuropathy, bone disease, sarcopenia, and frailty. Related books and key specific meeting abstracts were also taken into account.

## 3. Crucial Aspects to Consider in the Physiotherapeutic Management of Patients with AL or MM

### 3.1. The Importance of Exercise and Nutrition

Exercise is a mainstay of PT. It can be prescribed and applied in many ways to a myriad of clinical scenarios, including frailty prevention and whenever possible, frailty reversion [32]. A systematic review focusing on older and frail general patients (without AL), covering 36 trials, showed that structured physical-activity (PA)-based PT reduced frailty and enhanced physical, cognitive and emotional resilience [33]. However, most studies on the impact of exercise-based interventions in amyloidosis are focused on transthyretin (ATTR)-CA, whereas those restricted to AL-CA are very limited. No specific evidence-based guideline for the PT assessment and exercise-based management in AL patients is available so far.

The impact of “geriatric conditions”, particularly frailty (not necessarily geriatric in the AL setting) [25,34], but also comorbidity [35], malnutrition [36], sarcopenia [37], cognitive impairment and mood disorders [38,39], unwanted personal and social loneliness [40], polypharmacy [41], and more, impact prognosis and other key issues such as optimal self-care and MDT-based clinical decision making [24]. Despite the above complexity, the geriatric assessment and management in older adults with cancer is overall cost-effective, although future research should identify optimal core components and patient selection criteria [42].

As a rule, exercise training (ET) tolerance is impaired in AL-CA. Moreover, older and frail AL patients (there is a strong association between frailty and HF) have a dismal prognosis [25], largely due to AL-CA-associated HF and arrhythmia. The PT in charge should evaluate the specific clinical context, perform standardized tests to measure mobility at baseline, ascertain therapeutic goals, and reassess periodically to optimize therapy, keeping in mind a practical, coordinated, comprehensive, and tailored approach. In a retrospective single-center study from a referral center with MDT and comprehensive amyloidosis clinic (CAC), 64 amyloidosis patients (only 16 AL) were evaluated for functional impairment, showing that patients with CA and particularly those with AL had significantly impaired functional mobility [43]. A tailored program of exercise should be incorporated in the supportive care for AL patients [44] but only QEPs should be responsible. However, QEPs remain an underutilized resource even in modern and developed healthcare systems [29], highlighting the need for coordinated actions to standardize this changing field so that exercise can reach its well deserved place in every healthcare system.

In contrast to the striking lack of data on AL-focused exercise programs, the landscape is extremely more flowery in MM. Moreover, the importance of ET has also been pointed out in the context of precursor diseases, such as monoclonal gammopathy of uncertain significance and smoldering MM [45]. Evidence-based guidelines have been proposed for the PT management of patients with MM [46,47]. Several recent studies focus on ET in MM [48,49,50,51], including clinical trials and systematic reviews [52,53,54,55,56,57]. Overall, ET in MM patients is safe and feasible. Most studies show an improvement in physical function and HRQoL, mainly due to a decrease in fatigue and pain. Interestingly, a study highlighted that ET was also able to increase the percentage of activated T lymphocytes in the bone marrow microenvironment, a change that may help to control the clonal cell proliferation [58]. If this translates into a better response and survival remains to be demonstrated.

Additionally, aerobic exercise has demonstrated a positive impact in older adults with mild cognitive impairment [59], although its specific effect in MM remains to be determined. In a recent scoping review, the most frequently used types of exercise were aerobic exercise, resistance training, and Nordic walking [60]. A growing body of evidence supports including ET in supportive care guidelines for MM. However, a relatively old guideline did not mention this approach [61]. Fortunately, the role of ET is highlighted in more recent specific guidelines [62]. Referral for a tailored ET program should be considered in the treatment of every NDMM patient, and reassessed accordingly throughout the changing clinical course.

Nutrition and PA are closely linked and must be adapted to the clinical situation [63,64,65,66,67].

Despite increasing evidence, cancer-related malnutrition is under-recognized and under-treated in the real world (RW) [68]. Therefore, there is room for improvement, and PTs should frame their interventions in coordination with nutritionists and allied professions.

Nutrition and PA are addressed together in key MM guidelines [69], but full adherence to these recommendations is remarkably low [70]. Providing specific dietary recommendations for MM patients is a complex task [71], considering the above-mentioned heterogeneity and diversity of changing clinical scenarios. Published and ongoing exercise and diet studies in MM were recently reviewed [72], emphasizing the need for more comprehensive and well powered studies on this challenging field. Interestingly, healthier pre-diagnosis dietary habits in MM patients were associated with longer OS [73]. A recent survey of 299 MM patients highlighted the relevance of dietary advice [74]. Several nutritional scores have been used to predict the outcome in NDMM patients [75,76]. Some are very simple such as the nutritional risk index (based on weight and albumin) whereas others are more complex such as the inflammatory and nutritional scoring system. The Prognostic Nutritional Index (PNI) as an independent prognostic factor in terms of OS following ASCT has been underlined in a study involving 245 MM patients [77]. A recent meta-analysis including 1120 MM patients confirmed that a lower PNI was associated with poorer progression-free survival (PFS) and OS [78]. Patients with both low PNI or high Controlling Nutritional Status (CONUT) index exhibited higher OS in relapsed or refractory (RR) MM (RRMM) patients receiving chimeric antigen receptor (CAR) T-cell therapy [79]. However, pre-transplant indices were unable to predict the risk of pneumonia or one-year mortality post-ASCT in MM [80].

ASCT remains a standard procedure in both AL and MM, with a high impact on PA and nutrition. With current selection criteria [81], this procedure is feasible and safe in about 25% of AL patients [17]. It is commonly performed as consolidation therapy in NDAL but it can also be an option in RRAL, provided that patients meet the above criteria. Transplant-related mortality (TRM) in AL is similar to that in MM, approaching 0% in many recent series. However, patients should be as fit as possible at the time of transplantation to improve their tolerance and avoid complications. For this reason, several studies emphasize the importance of nutrition and exercise during this stage. Malnutrition during the ASCT procedure is a poor prognostic factor in terms of clinical outcome, response to therapy, QoL, and cost. Moreover, nutritional care is heterogeneous among centers, and critical issues in the field have been defined to maximize outcomes [82]. Nutritional prehabilitation interventions can mitigate post-transplant malnutrition and improve ASCT outcomes [83]. However, information about PT-led ET in AL patients during the procedure is lacking, contrary to what happens in MM. Regarding MM, despite recent advances in the anti-clonal therapy in MM and inconsistent definitions of transplant ineligibility [84], ASCT remains a cornerstone for TE patients due to a favorable clinical risk/benefit ratio. However, several risks are increased during the procedure including drug toxicities, thrombosis, infection, malnutrition, and mortality. A risk-adapted, supervised, and tailored exercise program is crucial to maintain HRQL and minimize complications. Recent RW studies and clinical trials explore the feasibility, adherence, and clinical benefit of structured prehabilitation and rehabilitation exercise programs in MM patients undergoing ASCT [85,86,87,88,89].

### 3.2. Physiotherapy Interventions According to the Pattern of Key Organ Involvement

#### 3.2.1. Heart

The relevance of AL-CA is paramount for AL or AL/MM patients. Updated guidelines can help to standardize HF therapy [90,91]. A consensus statement for the assessment and management of frailty in advanced HF has been developed [92]. The composition and coordination of a comprehensive MDT for CA care patients has been proposed [93]. The New York Heart Association (NYHA) functional class is an easy score to classify patients in accordance with their heart-related PA limitations, ranging from no limitation (class I) to the presence of symptoms (fatigue, dyspnoea, palpitations, angina) even at rest (class IV). Cardiopulmonary exercise testing (CPET) is a functional test able to assess the cardiocirculatory, pulmonary, and muscular systems during standardized exercise. CPET has a critical role in the study of HF associated with CA and may facilitate a personalized approach based on individual dynamic patterns of performance [94]. PTs should be part of the MDT, particularly in CAC of referral centers, adapting their interventions to established cardiological protocols. ET is a recommended treatment for unselected HF patients. The ERICA study [95] will test ET in ATTR-CA using the distance obtained at the 6 min walk test (6MWT) as the primary end-point. No similar studies are available yet in AL-CA.

HF-unrelated fatigue is a poorly defined symptom with high impact on HRQoL [96]. In AL patients without CA or other causes of HF, a holistic approach is needed to elucidate potential causal factors, including anemia, stress, depression, sleep disorders, physical inactivity, drug toxicity, and others. A tailored and progressive exercise program may be helpful in many of the above situations. A recent scoping review summarized the fatigue assessment methods used by PTs [97]. No specific reference appears for AL, and only one for MM. This is probably due to the fact that fatigue in AL is mostly secondary to HF and the evolutive control may be based on the HYHA score and serum cardiac biomarkers. The most frequently used scale in cancer was the European Organisation for the Research and Treatment of Cancer Quality of Life Questionnaire Core-30 (EORTC QLQ-C-30). HF-unrelated fatigue is also very prevalent in patients with MM, and may be the main symptom [98]. The underlying cause or causes should be identified and treated, given their enormous impact on HRQoL. It must be kept in mind that causes of fatigue in AL and MM are largely similar, and that the coexistence of both entities is not a rare event. Therapy should always be included as potential cause of fatigue. In contrast with AL, in MM the use of drugs such as immunomodulatory drugs, specifically lenalidomide, is a common cause of fatigue. Again, a multidisciplinary approach, including coordinated PT-led interventions, is key to maintaining and improving HRQoL. Strategies should focus on personalized and supervised PA/ET, an adapted and balanced diet, and emotional support.

#### 3.2.2. Kidney

The amyloid deposition in the kidneys commonly leads to nephrotic syndrome and/or chronic kidney disease (CKD) and sometimes end-stage renal disease (ESRD). PT is crucial in CKD patients [99]. ET should be included in the routine management of patients on hemodialysis (HD) [100]. Recent guidelines summarize the role of ET in CKD patients [101,102]. Although the benefit of ET is well established in the CKD setting, its level of implementation is still low even in higher-income countries [103,104,105] due to several potentially manageable barriers that should be overcome to bridge the current gap. A case report suggests the potential benefit of pre-transplant exercise in AL complicated with nephrotic syndrome [106].

Renal impairment is common in MM. Rapid intervention to reverse renal dysfunction is critical for the management of NDMM patients [107,108]. MM patients with CKD and particularly those on HD must follow ET general recommendations on the subject [99,100,101,102]. The outcome of NDMM patients requiring HD remains poor, although almost half of them can achieve dialysis independence and this group has a significantly better survival [109]. Individualized low-protein diet in MM patients with renal impairment showed benefits in renal function, nutrition, and HRQoL, being a simple, feasible and effective strategy [110].

#### 3.2.3. Bone

Bone disease is a hallmark of MM. Bone pain is common in MM and is primarily located in the spine. Osteolytic lesions are present in most NDMM patients, increasing the risk of skeletal-related events (SREs), mainly pathologic fractures and spinal cord compression [111]. SREs have a great impact on survival, HRQL, and costs [112,113,114,115]. Every effort should be made to treat these complications early and, whenever possible, prevent them with standardized medical and surgical approaches. This applies to MM but also to other precursor diseases [116,117,118,119,120]. The role of PT in the event of specific fractures or spinal cord compression is well documented, but the main focus of PT should be directed towards the prevention of these complications, using tailored ET programs and other complementary interventions [121]. A recent meta-analysis exploring non-pharmacological interventions on bone health among patients with low bone mass showed the protective effect of exercise on lumbar spine and femoral neck bone mineral density [122]. The impact of a PA-based approach on the outcome of MM should be graded and standardized to refine future PA-specific guidelines on this subject [123]. Moreover, a risk-based approach for the implementation of ET in MM is mandatory, due to the clinical heterogeneity of MM and the lack of MM-specific ET guidelines [124]. The role of baseline and dynamic frailty assessment is crucial in this regard.

#### 3.2.4. Nerve

Autonomic and peripheral neuropathy (PN) are common manifestations in AL patients, with an incidence rate of about 20–35% [125,126]. Key neurological manifestations in AL are PN, carpal tunnel syndrome (CTS), lumbar spinal stenosis, and amyloid myopathy.

AL-associated PN is commonly displayed as a length-dependent, sensory-predominant neuropathy, sometimes associated with autonomic failure (gastroparesis, orthostatic hypotension, diarrhea or constipation, or impotence) [127]. Numbness, paresthesia, motor deficits, and pain are common symptoms. This picture can be present as an initial presentation [128]. The intensity of the PN is usually higher in AL than in MM patients [129]. Pain reduction is a crucial goal of PT. Pharmacological treatment for PN-associated pain has limited effect. Exercise is effective in neuropathic pain through diverse mechanisms [130,131]. CTS is the most common nerve entrapment disorder worldwide, with a prevalence of about 5% in the general population [132]. Compression of the median nerve (uni- or bilateral) may lead to tingling and numbness of the thumb and index, impaired dexterity, weakness and pain, seriously compromising HRQoL. The etiology is multifactorial, but bilateral CTS can be an early sign of amyloidosis and therefore, CA should be excluded mainly in patients with bilateral CTS without occupational risk factors [133]. The management of CTS involves non-surgical (PT, corticosteroids, local injection, splinting) or surgical (open or endoscopic) interventions, all aimed at restoring function and alleviating pain. Amyloid myopathy is due to amyloid deposition in muscle tissue. It is mainly characterized by proximal muscle weakness in the limb-girdle distribution, but dysphagia, myalgia, macroglossia, jaw claudication, and other symptoms may be present. This is a rare but probably underdiagnosed event [134,135].

PN may be associated with any of the entities that present with monoclonal gammopathy (MG) [136]. MG-associated PN is often a difficult diagnosis with limited treatment options [137]. One third of NDMM patients may have abnormalities on electrophysiologic studies (axonal damage in typical cases), but PN is present in about 10%, sometimes preceding the diagnosis of MM [138]. Weakness and numbness of distal limbs is the most common clinical presentation. The information about the role of autonomic nervous system is scarce and controversial. However, chronic stress and sympathetic activation have been associated with increased mortality in MM [139]. POEMS (PN, organomegaly, endocrinopathy, M protein, skin changes) is a paraneoplastic syndrome that can be associated with a PCN [140]. In contrast to MM, bone lesions are sclerotic. The PN is motor predominant, frequently with associated pain and sensory symptoms. Guillain–Barré syndrome (GBS) is an acute immune-mediated heterogeneous PN characterized by rapidly progressive bilateral weakness that can be triggered by infection, vaccination, surgery and some drugs. There are a few reported MM cases that developed GBS after using bortezomib [141,142]. Regarding PN in MM, the aim of PT is maintaining physical function and HRQoL. Preventing falls is crucial to avoid fractures. The first line of treatment for NDMM patients includes proteasome inhibitors such as bortezomib, and immunomodulatory drugs such as lenalidomide. Both groups of drugs can cause sensory and motor nerve damage [143,144,145].

Transplant-related neuropathies and myopathies may occur during the procedure [146]. Neuralgic amyotrophy is a rare and under-recognized peripheral nerve disorder characterized by sudden shoulder pain followed by weakness in patients with hematological neoplasms, including MM. Magnetic resonance imaging and nerve studies confirm the diagnosis. The management is supportive, focusing on pain control and rehabilitation. A retrospective series of nine patients has been reported [147].

#### 3.2.5. Muscle, Sarcopenia

Sarcopenia is a progressive, generalized, and accelerated loss of skeletal muscle mass and function leading to decline in physical function and mobility as well as to increased risk of adverse outcomes including falls, fractures, and premature mortality [37]. Sarcopenia may be classified as primary (when aging is the only evident cause) or secondary. Early identification of cancer-related computed tomography (CT)-defined muscle loss is essential to enable timely interventions and mitigate adverse outcomes. A total of 13 factors have been consistently associated pointing out patients requiring timely referral for nutrition assessment and ET interventions [148]. RW daily clinical practice shows a high incidence of sarcopenia in older and frail NDAL patients. This population has a dismal prognosis and represents a global challenge, including a diet-related poor nutritional status [149].

MM-associated secondary sarcopenia predominantly affects older and frail patients. In a recent review, key studies on MM-associated sarcopenia were analyzed, most of them in the NDMM setting [150]. The study underscored the need for a comprehensive, holistic, and standardized geriatric assessment as a standard-of-care tool. Resistance training is the first-line treatment to improve sarcopenia, but aging negatively impacts a significant improvement of muscle mass [151]. An integrated approach including resistance training, anti-inflammatory nutrition and targeted supplements improves muscle strength [152].

## 4. Discussion

AL and MM are two different, but closely related entities. Both are complex, heterogeneous, chronic, and incurable diseases, included in the wide group of monoclonal gammopathies. Both share similar anti-clonal therapy, including ASCT in selected TE patients. The main difference is highlighted by the epidemiological background. AL is a rare disease, whereas the incidence of MM is overall five times higher, being the second most common hematological neoplasm. Another key distinction is the clinical scenario. In AL the heart is the most frequently involved organ and the extension of cardiac involvement is the main prognostic factor. On the contrary, the clinical picture in MM is primarily marked by bone disease. Significantly, OS is steadily improving in both cases, and consequently the prevalence is increasing, and with it the associated health burden. However, HRQoL remains a concern, particularly in older patients in whom the clinical impact of the disease is compounded by various age-related factors such as frailty, disability, and comorbidity. Additionally, the preferred pharmacological regimens for ND- or RR- AL or MM have changed over the past two decades [153,154], and their corresponding toxicity profile. The right treatment should be applied to the right patient at the right moment. Feasibility, availability, efficacy, safety, adherence, and patient consent should be assessed. Guidelines are of great help, but patient-associated nuances and circumstances may challenge even the best up-to-date guidance. Every single patient at every single moment is unique. A patient may be considered frail one day and fit enough for a certain procedure three months later. Therefore, the indication and schedule of an intervention should be adapted accordingly. The complexity of AL should be addressed through a comprehensive and holistic approach including epidemiological, diagnostic, prognostic, therapeutic and management issues [155].

Clinical decision making should always be based on an evidence-based, dynamic, and personalized risk/benefit approach, and should be shared with patients and caregivers. For instance, the indication of performing ASCT in AL is based on strict inclusion and exclusion criteria to select the patients who can safely benefit most from the procedure. Obviously, the lower the incidence, the less weight the available evidence. Consequently, modern guidelines show an evidence-graded approach for each recommendation. The available information for MM in each of the above-mentioned items is much greater than for AL, for which there is hardly any evidence. The need for clinical decision making and the limitation in the quantity and quality of information in some areas should be translated to the PT arena.

**Figure 1:**
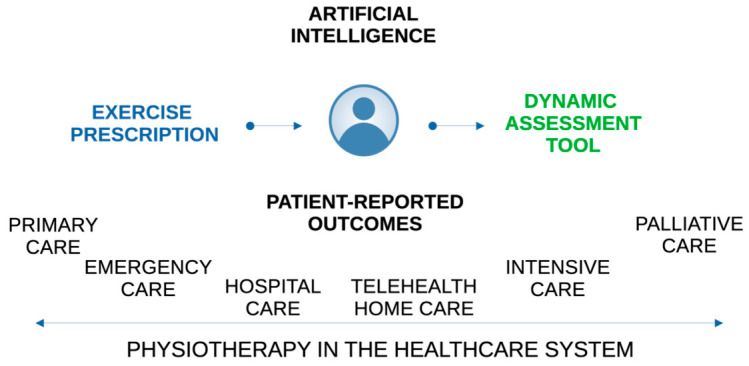
The scope of the physiotherapeutic interventions.

The PT intervention should follow a standardized and practical approach. First is evaluation of the clinical context of the patient, summarized in Table 1. The scope of PT intervention covers the entire spectrum of the healthcare system (Figure 1): primary care [156,157,158,159], hospital care [160,161], intensive care [9,162,163], emergency care [8,164], palliative care [165,166], home care [167,168], tele-healthcare [169], or mixed models [170]. Disparities between the available resources in different healthcare systems and different areas of the same healthcare may influence the type of care or the need to transfer the patient [171]. Basic clinical information should be available in the electronic medical record. Mandatory variables to consider are the following: department or person of reference requesting the intervention, level of urgency required and reason, patient location, type of healthcare (private, public), native language, age, sex, Eastern Cooperative Oncology Group (ECOG) performance status (PS), weight, height, BMI, comorbidities [172], staging (usually Revised International Staging System for MM and the 2025 AL-International Staging System for AL), basic clinical laboratory data, imaging (particularly the presence of osteolytic lesions, fractures, or extramedullary disease in MM), and current therapy, including active participation in clinical trials. If available, scores on HRQoL, nutrition and frailty are of great interest. Second, there is a tailored interview and physical exploration. Third is prescription. Selection of the intervention program, schedule, tool to assess the planned benefit, and baseline assessment. Patient consent and preferences should also be taken into account. Fourth, follow-up. Complementary interventions could also be eventually planned as well as education tips and lifestyle advice [173].

**Figure 2:**
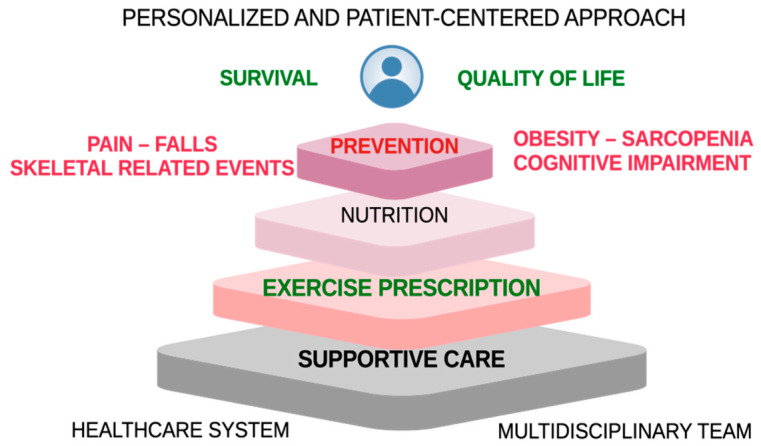
Exercise prescription as the key physiotherapy intervention.

Exercise is the main PT intervention. It represents a critical component of the supportive care and has a primarily preventive objective (Figure 2). The different types of exercises are described in specific reference sources [174]. Cumulative evidence in AL and particularly MM shows that ET is safe and effective in most cases, with a positive impact on HRQoL. The indication, type of exercise, type of supervision, duration, conditions, objective PA, and schedule should be recorded in the electronic medical record. In summary, a personalized exercise prescription should be issued [175,176,177]. As such, exercise-related severe adverse events should be recorded separately. Adherence to exercise prescription is a critical issue. Remarkably, implementing exercise in obese patients is challenging. In patients with MM, a significantly negative correlation between adherence and BMI was demonstrated [178]. Strategies to promote exercise adherence in obese people have been developed but new and robust studies are needed to progress on this subject [179].

Different methods are used to evaluate the clinical benefit of the exercise intervention in clinical trials, such as functional tests (6MWT, timed sit-to-stand, hand grip strength, and more) and HRQoL questionnaires. The information relative to exercise prescription and the selected tool for evaluation is commonly well described in clinical trials. Similarly, this information should also be available in the RW setting whenever possible.

Gaps remain between optimal or ideal care and RW daily clinical practice. The participation of PTs in an MDT/CAC is strongly recommended to optimize the outcome of patients. Healthcare managers should be informed of advantages of this approach, a model of excellence implemented in referral centers. Coordination with the community hospital and primary care is crucial. Despite recent advances and cumulative evidence, the role of PTs seems to be underutilized due to several barriers [180].

New strategies are needed to bridge the gaps (Table 2). Beyond HRQoL, patient-reported outcomes (PROs) are validated tools that inform patients’ symptoms, functional status and HRQoL in ePRO platforms, helping in the real-time symptoms monitoring and dynamic clinical decision making [181]. As expected, the use of PROs in AL is limited [182]. The most frequently used instruments have not yet been validated in AL. The current lack of disease-specific instruments and standardization is a surmountable obstacle. The use of PROs in MM is well developed in clinical trials but there is room for improvement in some methodological issues [183,184]. Moreover, PROs are time-consuming, hindering their use in the RW setting. A 3-item questionnaire for fatigue, pain, and HRQoL was incorporated at Mayo Clinic, showing that patient-reported symptoms have independent prognostic value in NDMM [185]. Importantly, the role of caregivers in AL and MM is increasingly emphasized. Therefore, caregiver-reported outcomes or family-reported outcomes are also assessed [186,187]. Patient education plays a critical role in the modern management of AL and MM [188]. Remarkably, pain control is crucial particularly in MM. Integrating PROs into exercise prescription will allow for a more precise and personalized treatment and assessment [189].

The types of PT interventions are also evolving. Given the growing waitlists and costs, group-based PT interventions have demonstrated similar adherence to exercises and comparable clinical effectiveness to personal care in several conditions [190]. Some AL and MM patients have mild cognitive impairment for different causes. These patients can benefit from music therapy as a complementary approach [191,192]. Dance may offer a similar effect [193] as well as virtual reality-based interventions [194]. Mind–body exercises (hypnosis, yoga, tai chi, Qigong) may improve physical performance, HRQoL, and mood disorders in cancer patients [195], but specific information for AL and MM patients is limited. Patients with pain and musculoskeletal disorders unable to tolerate exercise-based rehabilitation may benefit from laser acupuncture [196], but its impact on AL and MM remains to be determined.

Table 3 summarizes some critical differences in physical therapy interventions according to the three main clinical scenarios (AL, MM, and AL/MM), which reflect a specific pattern of organ involvement. The comorbidity pattern of each patient should be taken into account to offer a tailored ET prescription. Whenever possible, a frailty score should be dynamically used throughout the course of the disease.

PT-guided exercise prescription apps with accredited scientific evaluation can be a valuable tool to support the remote delivery of the exercise program, but some issues such as guidance on the progression and tailored adjustment should be improved [197]. Exercise should be increasingly viewed as “medicine” [198].

AI is progressively influencing the diagnosis, prognosis, and treatment of AL and MM patients, aiming to improve the global streamline workflow efficiency and to enhance precision and personalized care. Exercise interventions are also being refined according to AI-driven advances, improving treatment response prediction. However, gaps and barriers remain in this exciting and rapidly changing field [199,200,201,202,203,204,205,206,207,208].

The PT research agenda is exciting and challenging [11,12,13,209]. An evidence-based approach should always be applied to update specific supportive care guidelines and translate advances in clinical trials to the RW setting, in a highly complex clinical and technological changing scenario. Unmet clinical needs should be identified as well as areas of uncertainty or controversy, particularly those associated with negative impact on the outcome. Consequently, strategies to prevent or reverse obesity, frailty, disability, HF, malnutrition, osteopenia-associated SRE, sarcopenia, infection, social isolation, and financial toxicity should be undertaken. Moreover, PT practice should always be adapted to the social network and community background of every single patient. A shared, standardized, and efficient use of electronic health records is crucial to undertake observational RW and cost-effectiveness studies [210]. Boosting PT research with high-quality clinical trials is mandatory to ensure efficiency and the best clinical results. Whenever possible, a clinical trial should always be the first option of treatment.

Finally, a large dose of optimism should be applied whenever possible at every step [211]. Although AL and MM are not currently preventable diseases, the concept of cancer prevention should always be underlined. A significant percentage of cancer in general is preventable with simple measures [212]. Three out of the fourteen key ways identified to prevent cancer focused on PA, obesity, and diet.

## 5. Conclusions

Supportive care is an essential part of the treatment for AL and MM patients. Exercise is a key component of supportive care. Overall, exercise is considered feasible, safe and valuable to improve HRQoL in cancer patients in general. PT-led tailored exercise programs are also considered safe and effective in AL and MM, improving HRQoL in most studies. However, some barriers and knowledge gaps remain. The specific evidence for AL is scarce due to the difficulty in conducting studies because of its low incidence, and the outcome is largely conditioned by the intensity of cardiac involvement. The cumulative evidence in MM is stronger. However, the comprehensive role of PT in the care of AL and MM patients is not fully integrated as standard of care in higher-income countries, with the exception of MDT in referral centers. A collective and coordinated effort should be made between all implicated healthcare professionals to integrate exercise prescription in future specific, coordinated, and comprehensive guidelines for AL and MM. Alternatively, established evidence-based guidelines for AL and MM could be enriched including updated information on the global role of PT interventions through the complex clinical course of both entities.

Standardized exercise prescriptions and specific outcome validated tools should be used in AL and MM patients. PROs should be incorporated in RW exercise interventions. Pain, frailty, nutrition, and HRQoL scores should be dynamically used in AL and MM patients through their complete evolutive course. Future research must focus on prevention and should be based on AI-driven advances and large-scale multi-center clinical trials, which are particularly necessary in rare diseases such as AL.

## Figures and Tables

**Table 1:** Clinical information needed before first physiotherapist intervention in systemic light-chain (AL) amyloidosis and multiple myeloma patients.

Scope of healthcare	M
Type of healthcare	M
Requesting person	M
Level of urgency	M
Clinical target	M
Patient location	M
Native language	M
Age	M
Sex	M
Weight, height, BMI	M
ECOG-PS	M
Comorbidities	M
Staging	M
Hemoglobin	M
Serum calcium	M
e-GFR	M
Imaging	M
Current therapy	M
Clinical trial	M
Six-minute walk test	R
HRQoL	R
Nutrition score	R
Frailty score	R

BMI: body mass index; ECOG-PS: Eastern Cooperative Oncology Group (ECOG) performance status (PS); e-GFR: estimated glomerular filtration rate; HRQoL: health-related quality of life; M: mandatory; R: recommended.

**Table 2:** Bridging gaps for excellence: Physiotherapy integration in the real-world healthcare for systemic light-chain (AL) amyloidosis and multiple myeloma patients.

Future	Present	Issues
Full spectrum	Hospital-based	Scope
Multidisciplinary unit	Department	Setup
Prevention	Treatment	Focus
Holistic AI-based	Clinical	Approach
On-site	On demand	Imaging
Standardization	Heterogeneity	Baseline study
Dynamic	Variable	Follow-up
Integrated	Recommended	Geriatric assessment
Systematic	Punctual	Frailty score
Systematic	Punctual	Nutritional score
Standardized	Variable	Comorbidity
PROs, e-platforms	Old tools	HRQoL
Real-time tele-health	Physical visit	Patient–staff interaction
Personalized	Adapted	Exercise prescription
Standardized	Variable	Outcome measures
Collaborative trials	Limited	AL Research
Optimized	Heterogeneity	Contextualized PT
Strong	Weak	Patient education
High value	Low value	Research in PT cv

**Table 3:** Main differences in physiotherapy interventions according to the clinical scenario.

AL/MM	MM	AL
Extended ET adaptation	Anemia and bone adapted ET	CA-AL adapted ET
Mixed	Focus on pain and SREs	Focus on HF and arrhythmias
Mixed	Aerobic exercise, resistance training, Nordic walking Avoid high-impact activities and end-range movements	Functional tests (CPET, 6MWT, NYHA)- tailored ET
Mixed	Bone imaging tailored	Cardiac imaging tailored

AL: systemic light-chain amyloidosis; CA-AL: cardiac involvement in AL amyloidosis; CPET: cardiopulmonary ET; ET: exercise training; HF: heart failure; MM: multiple myeloma; 6MWT: 6 min walk test; NYHA: New York Heart Association functional class; and SREs: skeletal-related event.

## Data Availability

No new data were created or analyzed in this study.
